# Secondary care usage and characteristics of hospital inpatients referred to a UK homeless health team: a retrospective service evaluation

**DOI:** 10.1186/s12913-019-4620-1

**Published:** 2019-11-21

**Authors:** Hannah Field, Briony Hudson, Nigel Hewett, Zana Khan

**Affiliations:** 1 Royal Surrey County Hospital, Egerton Road, Guildford, GU2 7XX UK; 2 Pathway, 250 Euston Road, London, NW1 2PG UK; 3 Marie Curie Palliative Care Research Department, Division of Psychiatry, University College London, 6th Floor Maple House, 149 Tottenham Court Road, London, W1T 7NF UK; 4 UCL Collaborative centre for inclusion health, Department of Primary Care and Population Health, University College London, London, UK

**Keywords:** Homeless person, Patient readmission, Health services research, Cost savings, Inclusion health, Pathway, Hospital medicine, Retrospective study

## Abstract

**Background:**

UK “Pathway” teams offer specialist hospital care coordination for people experiencing homelessness. Emergency healthcare use is high among homeless people, yet “homelessness” is not routinely coded in National Health Service (NHS) data. Pathway team records provide an opportunity to assess patterns in admissions and outcomes for inpatients identified as homeless.

**Methods:**

Retrospective analysis of patients referred to “Pathway” homelessness teams in seven UK hospitals to explore the patterns of hospital admission, morbidity, secondary healthcare utilisation and housing status. Each patient was individually identified as experiencing homelessness. Within a six-month period, demographic data, reason for admission, morbidity, mortality and secondary care hospital usage 120-days before and 120-days after the index admission was collected.

**Results:**

A total of 1009 patients were referred, resulting in 1135 admissions. Most admissions had an acute physical health need (94.9%). Co-morbid mental illness and/or substance misuse was common (55.7%). Reasons for admission included mental and behavioral disorders (overdose, alcohol withdrawal or depression, 28.3%), external causes of morbidity and mortality (assault or trauma, 18.7%), and injury, poisoning and external causes (head injury, falls and fractures, 12.4%). Unplanned Emergency Department attendances reduced after index admission and unplanned hospital admissions increased slightly. Planned admissions doubled and total bed days increased. Housing status was maintained or improved for over 60% of inpatients upon discharge. Within 12 months of index admission, 50 patients (5%) died, 15 deaths (30%) occurred during the index admission.

**Conclusions:**

Disengagement with health services is common among homeless people. Many deaths are due to treatable medical conditions (heart disease, pneumonia, cancer). Observed increases in planned admissions suggests intervention from Pathway teams facilitates necessary investigations and treatment for homeless people. Equity, parity of care, and value should be inbuilt interventions for inclusion health groups and evaluations need to move beyond simply seeking cost reductions.

## Background

### What is already known on this topic

Recent international evidence reviews define Inclusion Health Groups (IHGs) as overlapping populations including those experiencing homelessness, prisoners, people who sell sex and people with substance use disorders [1, 2]. IHGs experience extreme health inequity, high morbidity and premature mortality [2]. Due to barriers such as stigma and difficulty making and keeping appointments, use of primary care services are low for IHG’s, while emergency healthcare use is high, often due to a healthcare crisis [3]. Duration of admission has been estimated to be three times longer for homeless patients who often experience poor hospital discharge arrangements [3]. This is likely to reflect ongoing and unaddressed care and housing needs [4]. All IHGs have frequent contacts with services, including emergency hospital attendances, but under-utilise scheduled and primary care due to a combination of chaotic lifestyles and barriers to registering and attending appointment based services [5–7]. This pattern of care focuses on addressing one problem, but fails to address a person’s broader health and care needs. This results in missed opportunities, poor health outcomes and significant costs to health and public services [8, 9].

Inclusion health (IH) describes an emerging movement which aims to prevent and redress the harms of extreme inequity among the most excluded populations through advocacy, policy, research, education, practice and service provision [1]. This agenda has encouraged the development of specialist primary and secondary healthcare and improving mainstream health and care provision for IHGs including people experiencing homelessness [1, 10–12].

### What this study adds

This study provides the first analysis of admissions for people referred to hospital-based homelessness teams across the UK. In this sample, patients experiencing homelessness were most commonly admitted for ICD-10 categories consistent with the life experiences of people living with poverty, deprivation and social exclusion, such as mental and behavioural disorders and external causes of morbidity and mortality. This study provides evidence for rethinking the definition of success for interventions aimed at improving the health of people experiencing homelessness. We suggest moving beyond seeking cost reductions to considerations of equity, parity of care and value [13, 14].

## Introduction

Homelessness takes many forms, living and sleeping on the street (rough-sleeping) remains the most visible form of homelessness, but as many as 170,000 people in Britain reside in other insecure settings [15] including homeless hostels, “sofa-surfing” (living temporarily with others), living in squats or other unsuitable and temporary accommodation such as bed and breakfasts [16]. The annual Government count of rough-sleepers estimates 4677 people slept rough on one night in Autumn 2018 [17] an increase of 165% since 2010 [18]. Research evidence suggests that the figure is actually around 24,000 including people sleeping in tents and cars [19]. These rapid increases in homelessness are underpinned by poverty and adverse life experiences but driven by austerity, welfare cuts, lack of affordable housing, and sustained cuts in the local authority services that support these groups [8, 20].

Homelessness is characterised by complex health and care needs often including “tri-morbidity” - the combination of physical illness, mental illness, and substance use disorders [3, 21–23]. A recent systematic review showed standardised mortality rates (SMRs) across IHGs are ten times that of the general population [2]. Although SMRs are highest for deaths from causes including overdoses, suicide, accidents and violence, they were also more than doubled for treatable conditions such as coronary heart disease, pneumonia and cancers, which account for the majority of deaths [2]. More secondary care for treatable conditions is a beneficial outcome for the individuals and for health equity, even if this results in increased costs.

The reported mean age of death for people experiencing homelessness is 42 for women and 44 for men [24, 25]. Barriers to accessing scheduled and primary care include perceived stigma and discrimination, difficulty making and keeping appointments [26], difficulty registering with a GP due to lack of identification and address [2], competing priorities [27], communication difficulties or challenging behaviour [8, 9, 28, 29]. In 2010, when homelessness was less prevalent, the estimated cost of unscheduled and emergency care was £85 million, but recent evidence shows that Emergency Department attendances (in the UK referred to as Accident and Emergency or A&E) of people experiencing homelessness have increased three fold since 2010/11 [30]. Therefore, secondary care has been one focus of interventions to improve care of people experiencing homelessness and other IHGs.

One model developed by the UK’s leading homeless healthcare charity, Pathway, includes specialist in-reach hospital care coordination teams, led by a specialist General Practitioner (GP), known as Pathway teams. These teams provide advocacy, advice and support both for the patient and for the admitting clinical team and liaise with community health and housing providers. Specialist GPs undertake hospital ward-rounds of homeless inpatients and are supported by senior clinical staff including nurses, social workers or occupational therapists (OTs), housing officers and Care Navigators (employees who have lived experience of homelessness and are best placed to support homeless inpatients) [10, 31, 32]. Several small observational studies have suggested a subsequent reduction in secondary care usage [10, 33, 34]. A two-centre randomised controlled trial did not confirm this finding but did show improved health and housing outcomes resulting in a cost effective intervention [35]. Pathway have trained ten hospital-based teams across the country, which operate as part of NHS services, this specialist care coordination for patients experiencing homelessness is hereafter called the “Pathway Intervention”.

While morbidity and mortality rates of people experiencing homelessness in primary care settings have been explored [36, 37], little research exists around the diagnostic reasons for hospital admissions for this group. Homelessness is not routinely coded in NHS data, making the identification of people experiencing homelessness within NHS records problematic. However, all the patients supported by Pathway teams are confirmed as homeless. We undertook a retrospective analysis of the records of Pathway homelessness teams across the UK to investigate morbidity patterns in this group and to look for any changes in secondary care usage after the Pathway intervention. We investigated:

- the recorded reasons for admission to hospital for patients seen by Pathway homelessness teams within a six-month period (1st January to 30th June 2016).
- secondary healthcare usage in the 120-days prior to and following this index admission and discharge.
- any change in housing status in the 120-days following index admission in comparison to housing status upon admission.

## Methods

### Study design and setting

A retrospective analysis of all admissions to secondary care involving Pathway teams located in hospitals in seven locations across the UK, over a six-month period (1st January to 30th June 2016). The medical records of identified patients were examined, and data extracted relating to secondary healthcare usage and housing status 120-days before their index admission and 120-days after discharge. Following an extensive search of the literature, there was no agreed methodology for this type of dataset and time-periods ranged from 30-days to 120-days before and after a defined intervention. The 120-day timeframe was selected to allow assessment of sustained discharge from hospital and capture a thorough picture of secondary care usage. Where available, the reasons for the index admission were gathered. Non-admitted patients (such as those seen in A&E or the community) and referrals not assessed by a Pathway team were excluded. The index admission and A&E attendance were not included in either the “before” or “after” analysis of secondary care usage to avoid weighting either data set.

Of the ten Pathway teams, seven were assessed covering: Bradford, Brighton, Manchester and four London based teams. The composition and size of Pathway teams vary according to the local scale of the challenge and available funding, but they all include GP leadership and a multidisciplinary approach. Excluded Pathway teams were Bristol (which was not in operation during the study period), Leeds (unable to visit due to team re-structuring) and the South London and Maudsley (which focuses on mental health).

### Study population

We assumed that all patients who had been referred and assessed by the Pathway teams were experiencing homelessness of some kind, as this was a requirement for engagement with these services. Individuals were identified by a unique identifier (medical record number). Data from patients referred, assessed and admitted to acute hospital trusts by Pathway teams between January and July 2016 were included.

### Data extraction

Patient data (clinical, demographic and admission related data relating to index admission) was retrospectively extracted from hospital records, hospital discharge summaries and Pathway databases within each study site and entered anonymously into an excel template by the researcher (HF). Data relating to admissions and A&E attendances 120-days prior to admission and 120-days following discharge was also extracted and the reason for attendance recorded. Housing status on admission and discharge was recorded, where available. Table 1 outlines all variables collected, and the sources from which this data was obtained. Each patient was assigned a unique code to maintain anonymity and no identifiable patient information was included. For data extraction template, see Additional file 1.

**Table 1:** Data extracted from Pathway database, hospital records and hospital discharge summaries

Outcome	Data source
Demographic characteristics	
Age during admission	Pathway database and hospital record
Gender	Pathway database and hospital record
Nationality/ recourse to public funds	Pathway database
Housing status	Pathway database
Clinical characteristics	
Primary reason for admission (ICD-10 code)	Hospital discharge summary
Secondary reason for admission (if applicable)	Hospital discharge summary
Multi-morbidity	Pathway database
Deaths (where applicable)	Hospital record
Admission characteristics	
Length of admission (days)	Hospital discharge summary
Type of admission (planned or unplanned)	Hospital discharge summary
Whether a surgery or procedure took place	Hospital discharge summary
Whether the admission was related to a recent trauma (road traffic accident, assault, overdose, other)	Hospital discharge summary and Pathway database
Whether drugs and/or alcohol were involved in circumstances of admission	Hospital discharge summary and Pathway database
Type of discharge (self-discharge or medical discharge)	Hospital discharge summary and Pathway database
Secondary care usage	
Readmission and A&E attendances 120-days prior to admission and 120-days following discharge	Hospital record
Characteristics of A&E attendances and admissions (length of admission, type of admission, reason for admission)	Hospital discharge summary

### Data analysis

The first admission for each patient occurring between 1st January 2016 and 30th June 2016 was identified as their “index admission.” This may or may not have been the first time that the patient had contact with the Pathway team. Each index admission was analysed independently, regardless of whether a patient re-attended in this timeframe. Frequent attenders were included to provide an accurate representation of patients presenting to the service. Length of stay was calculated by taking the date of admission from the date of discharge. For inpatients who died during the admission, date of death was used to calculate length of stay.

The International Classification of Diseases, tenth revision (ICD-10) [38] was used to categorise diagnostic reasons for admission provided. Each admission was allocated a primary diagnosis and, where present, a secondary diagnosis in order to capture the complexity of health need and prevalence of co-morbidities. ICD-10 diagnostic categories were summarised leading to a descriptive analysis of reasons for admission. Two researchers (HF and BH) categorised half of the data each using the ICD-10 online table and consensus was agreed amongst authors for coding any ambiguous diagnoses. The main reason for attending hospital was allocated as the primary diagnosis and another consequence of this was allocated the secondary diagnosis. The researchers then re-checked the coding of the entire data set and measures of agreement were calculated (k = .815, 95% CI, 0.01–1.62, *p* < .001 for the first 10% of the data) to ensure uniformity in categorisation.

Descriptive statistics were used to describe reasons for referral and secondary health care usage in the 120-days following discharge for each patient. Number of hospital attendances 120-days prior to the index admission were compared to those 120-days following discharge from the index admission using paired t-tests assuming continuous variables from independent observations with minimal outliers. Three types of hospital attendance were recorded: A&E attendance (presentation to the emergency department not requiring hospital admission), unplanned admission (unscheduled admission to hospital) and planned admission (scheduled admission for a procedure or treatment, organised in advance).

Change in housing status from admission to discharge was coded as either an improvement, deterioration or no change. An example of deterioration in housing status would be if a patient moved from private rented or hostel, to sofa-surfing, or from sofa-surfing or hostel, to rough-sleeping. An improvement would be assigned if the reverse were true.

### Missing data and handling

Missing data was encountered throughout data collection. Where percentages were calculated, the percentage of data missing was taken into consideration as a separate category for clarity, consequently other percentages may be an under-representation. Patients were not excluded if there was missing data for any of the variables collected. Repeat admissions and referrals to a Pathway team during the study period were included as a separate index admission in order to reflect the manner in which these patients use services, (this accounted for less than 10% of the patients referred).

### Ethics

As a service evaluation this study was exempt from ethical approval requirements [39]. An honorary contract was obtained for HF from each hospital trust for data collection. All data was fully anonymised prior to analysis.

## Results

### Number of referrals and admissions

Throughout January to July 2016, 1663 patients experiencing homelessness were referred across the seven Pathway teams. Almost one third of referrals were not admitted to hospital and were therefore excluded (*n* = 528, 31.7%). Almost 70% of referrals (*n* = 1135, 68.3%) resulted in hospital admissions and were included. These referrals comprised of 1009 (88.9%) individual patients. Ninety-one (9%) with 217 attendances to hospital were re-referred and re-assessed by a Pathway homeless team within the six-month period.

### Demographic characteristics

The average age at admission across the seven teams was 43 years (SD 22.8) (Table 2). Average age was consistent across the teams. Over three quarters (77%, *n* = 877) of patients were male. Male to female ratios were also consistent across teams. Seventy-five people (6.6%) were found to have “no recourse to public funds” (NRPF) which results in limited access to certain statutory services such as welfare benefits or housing from a local authority.

Regarding housing status, the largest group of patients (*n* = 473, 41.7%) were categorised as having no fixed abode or rough-sleeping. Almost a quarter of patients (*n* = 259) reported living in a hostel or temporary accommodation and just over 10% (*n* = 126) were living in council housing or a facility delivering medical care. One-hundred records had missing data for housing status (8.8%).

**Table 2:** Demographics and clinical characteristics of analysed admissions

Characteristic	Total (n)	%	Mean	SD
Number of referrals	1663	–		
Number of admissions	1135	100		
Number of patients	1009	88.9		
Average age on admission mean	–	–	43	22.8
Male gender	877	77.3		
Female gender	258	22.7		
Reported NRPF	75	6.6		
Reported self-discharge	45	4.0		
Reported deaths	50	4.4		
Average age of death mean	–	–	56	12.6
No fixed abode/rough-sleeping	473	41.7		
Unsuitable accommodation/sofa-surfing	177	15.6		
Hostel/ temporary accommodation	259	22.8		
Medical care/council house	126	11.1		
Unknown/missing data	100	8.8		

### Clinical characteristics

#### ICD-10 diagnoses

There was a broad spectrum of reasons patients experiencing homelessness were admitted to secondary care (Table 3). As well as alcohol related admissions such as pancreatitis, gastritis, decompensated alcoholic liver disease and cirrhosis, many patients were admitted for physical health problems unrelated to alcohol, such as deep vein thrombosis, myocardial infarction, pneumonia, sepsis and status epilepticus. Admissions were largely appropriate for acute hospital treatment. For full categorisation of diagnoses with ICD-10 codes, see Additional file 2.

**Table 3:** ICD-10 diagnostic categories with examples

ICD-10 Code	Example	Primary diagnosis *n* (%)	Secondary diagnosis *n* (%)	Total Prevalence
I Certain infectious and parasitic diseases	*HIV, tuberculosis, hepatitis B, hepatitis C, sepsis, meningitis*	54 (4.8)	16 (1.4)	70 (6.2)
I Neoplasms	*Cancer: lung, hepatocellular, GIST, renal cell, cervical*	30 (2.6)	8 (0.7)	38 (3.3)
III Diseases of the blood and blood-forming organs and certain disorders involving the immune mechanism	*Vaso-occlusive crisis, sickle cell, anaemia, pancytopenia, myelodysplastic syndrome*	12 (1.1)	5 (0.4)	17 (1.5)
IV Endocrine, nutritional and metabolic diseases	*Diabetic ketoacidosis, hyperglycaemic hyperosmolar state, hypokalaemia, SIADH, hyperglycaemia, hypoglycaemia, Wernicke’s encephalopathy, re-feeding syndrome*	39 (3.4)	45 (4.0)	84 (7.4)
V Mental and behavioural disorders	*Overdose, suicidal ideation, alcohol withdrawal, intoxication, self-harm, hallucinations, depression, bipolar, psychosis, schizophrenia*	210 (18.5)	111 (9.8)	321 (28.3)
VI Diseases of the nervous system	*Encephalitis, seizure, status epilepticus, multiple sclerosis*	74 (6.5)	5 (0.4)	79 (6.9)
VII Diseases of the eye and adnexa	*Glaucoma, visual disturbance, blindness, cataracts*	0 (0)	0 (0)	0 (0)
VIII Diseases of the ear and mastoid process	*Otitis media, otitis externa, perforated ear drum, Meniere’s disease*	0 (0)	0 (0)	0 (0)
IX Diseases of the circulatory system	*DVT, STEMI, leg ulcers, angina, PE, septic emboli, heart failure, palpitations, stroke, acute limb ischaemia, infective endocarditis, AF, oesophageal varices, amputation, aneurysm*	81 (7.1)	20 (1.8)	101 (8.9)
X Diseases of the respiratory system	*Pneumonia, T2RF, IECOPD, aspergilioma, pleural effusion, asthma*	83 (7.3)	35 (3.1)	118 (10.4)
XI Diseases of the digestive system	*Pancreatitis, peptic ulcer, gastritis, decompensated ALD, diverticulitis, oesophagitis, cirrhosis, cholecystitis, hernia*	92 (8.1)	14 (1.2)	106 (9.3)
XII Diseases of the skin and subcutaneous tissue	*Abscess, cellulitis, dermatitis*	78 (6.9)	15 (1.3)	93 (8.2)
XIII Diseases of the musculoskeletal system and connective tissue	*Osteomyelitis, septic arthritis, limb swelling, dislocation, back pain*	45 (4.0)	15 (1.3)	60 (5.3)
XIV Diseases of the genitourinary system	*UTI, CKD, Renal failure, urosepsis, pyelonephritis, renal stone*	37 (3.3)	8 (0.7)	45 (4.0)
XV Pregnancy, childbirth and the puerperium	*Caesarean, PPH, miscarriage, multiple gestation, obstetricdeath, disorders of pregnancy*	9 (0.8)	0 (0)	9 (0.8)
XVI Certain conditions originating in the perinatal period	*Birth trauma, perinatal infections, newborn disorders*	0 (0)	0 (0)	0 (0)
XVII Congenital malformations, deformations and chromosomal abnormalities	*Any congenital malformations, microcephaly, spina bifida, transposition of the great vessels, atrial septal defect.*	0 (0)	1 (0.1)	1 (0.1)
XVIII Symptoms, signs and abnormal clinical and laboratory findings, not elsewhere classified	*Unwell, collapse, abdominal pain, acute confusion, loss of consciousness, syncope, vomiting, diarrhoea*	84 (7.4)	29 (2.6)	113 (10.0)
XIX Injury, poisoning and certain other consequences of external causes	*Hypothermia, poisoning, head injury, stabbed, fracture, fall*	61 (5.4)	80 (7.0)	141 (12.4)
XX External causes of morbidity and mortality	*Road traffic collision, assault, head trauma, dog bite, deliberate self-harm*	89 (7.8)	123 (10.8)	212 (18.6)
XXI Factors influencing health status and contact with health services	*Ureteric stent, CABG, double cord transplant, cystoprostectomy, abortion*	20 (1.8)	57 (5.0)	77 (6.8)
XXII Codes for special purposes	*New diseases such as Zika virus, antimicrobial resistance*	1 (0.1)	0 (0)	1 (0.1)
Missing Data	*Data unavailable*	36 (3.2)	36 (3.2)	72 (6.4)

**Fig. 1:**
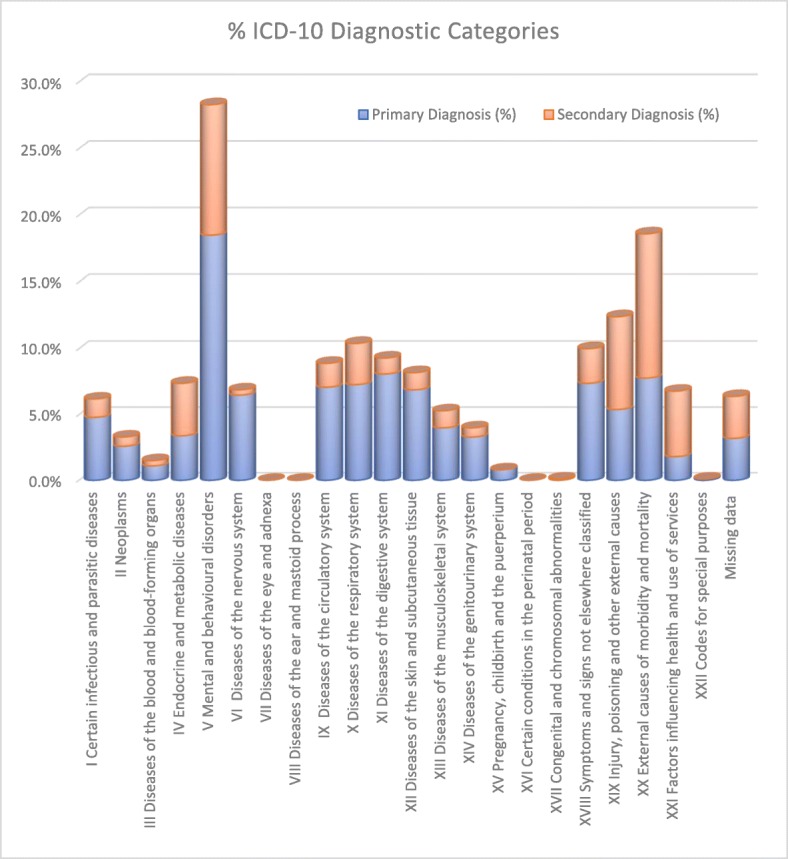
Representation of total primary and secondary care ICD-10 diagnoses

**Fig. 2:**
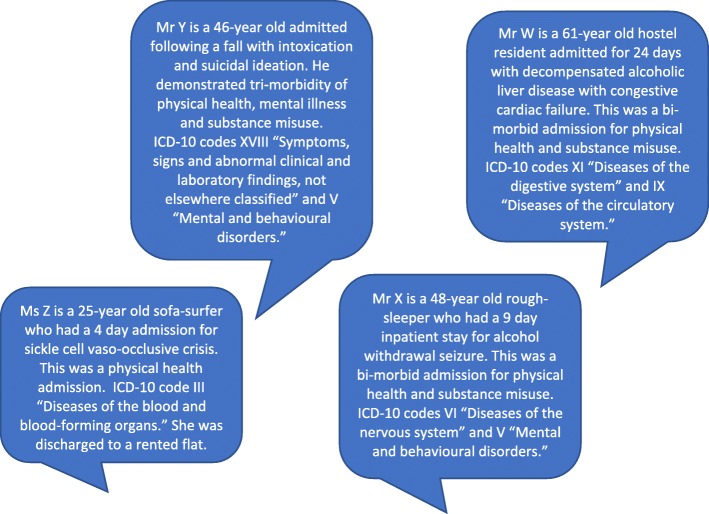
Case examples of analysed admitted inpatients experiencing homelessness

The most common ICD-10 category for both primary and secondary diagnosis was (V) Mental and Behavioral Disorders (28.3%) including overdose (6.9%), alcohol intoxication or withdrawal (8.0%), and suicidal ideation (1.9%) (Fig. 1). When taking into account primary and secondary diagnoses, (XX) External causes of morbidity and mortality made up 18.7% of admissions (including road traffic accidents (1.9%), assault (2.3%) and stabbing (1.5%)), (XIX) Injury, poisoning and certain other consequences of external causes made up 12.4% (fracture (5.6%), laceration (1.1%) and brain injury (3.5%)) and (XVIII) Symptoms, signs and abnormal clinical and laboratory findings not elsewhere classified accounted for 10.0% of admissions (collapse (2.0%), generally unwell (1.6%), pain (2.1%) and acute confusion (0.9%)). The primary and secondary categorised diagnoses are represented in Table 4. For full diagnostic characteristics, see Additional file 2. Examples of analysed admissions are included in Fig. 2.

**Table 4:** Classification of primary and secondary diagnostic ICD-10 in descending order. (*N/A* not applicable, *MD* missing data)

Primary Diagnosis			Secondary Diagnosis			Total Diagnoses		
ICD10	n	%	ICD10	n	%	ICD10	n	%
V	210	18.5%	*N/A*	512	45.1%	N/A	512	22.6%
XI	92	8.1%	XX	123	10.8%	V	321	14.2%
XX	89	7.8%	V	111	9.8%	XX	212	9.4%
XVIII	84	7.4%	XIX	80	7.0%	XIX	141	6.2%
X	83	7.3%	XXI	57	5.0%	X	118	5.2%
IX	81	7.1%	IV	45	4.0%	XVIII	113	5.0%
XII	78	6.9%	MD	36	3.2%	XI	106	4.7%
VI	74	6.5%	X	35	3.1%	IX	101	4.5%
XIX	61	5.4%	XVIII	29	2.6%	XII	93	4.1%
I	54	4.8%	IX	20	1.8%	IV	84	3.7%
XIII	45	4.0%	I	16	1.4%	VI	79	3.5%
IV	39	3.4%	XIII	15	1.3%	XXI	77	3.4%
XIV	37	3.3%	XII	15	1.3%	MD	72	3.2%
MD	36	3.2%	XI	14	1.2%	I	70	3.1%
II	30	2.6%	II	8	0.7%	XIII	60	2.7%
XXI	20	1.8%	XIV	8	0.7%	XIV	45	2.0%
III	12	1.1%	VI	5	0.4%	II	38	1.7%
XV	9	0.8%	III	5	0.4%	III	17	0.7%
XXII	1	0.1%	XVII	1	0.1%	XV	9	0.4%
VII	0	0.0%	VII	0	0.0%	XVII	1	0.0%
VIII	0	0.0%	VIII	0	0.0%	XXII	1	0.0%
XVI	0	0.0%	XV	0	0.0%	VII	0	0.0%
XVII	0	0.0%	XVI	0	0.0%	VIII	0	0.0%
N/A	0	0.0%	XXII	0	0.0%	XVI	0	0.0%

#### Multi-morbidity of admitted homeless patients

**Fig. 3:**
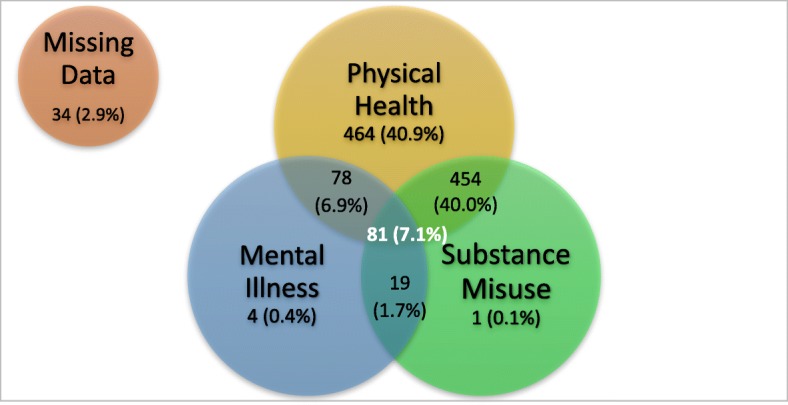
The tri-morbidities of homelessness

Almost 95% (*n* = 1077) of patients experiencing homelessness who were admitted to hospital had a physical health need either in isolation (40.9%) or in combination with mental illness (6.9%), addictions (40.0%), or both (7.1% of patients (*n* = 81) had documented tri-morbidity of physical health, mental health and substance use disorders). A small number of admissions were only related to mental illness (*n* = 4, 0.4%) or addiction (n = 1, 0.1%) and 19 (1.7%) patients were admitted for mental health needs combined with substance use disorders (Fig. 3). The number of admissions involving substance misuse or mental health issues should be interpreted with caution, as these were not reliably coded within the data reviewed.

### Mortality

Fifty reviewed patients (5%) died within one-year of discharge from their index admission (Table 5). The average age of death for these patients was 52 years. Of the patients that died, 30% (*n* = 15), died during their index admission, 16% (*n* = 8) died within 30-days of discharge and 20% (*n* = 10) between 30- and 120-days following discharge. More than a quarter (*n* = 14, 28%) died more than 120-days after discharge. The date of death was not reported for three patients (6%). Cause of death was only available for those that died during admission as it was not possible to access medical certificates for cause of death (MCCD). Of the 15 patients that died during their index admission, age of death ranged from 29 to 77 years. Over a quarter of these deaths (*n* = 4, 27%) were the result of trauma, three were cancer-related deaths, three were related to renal failure, two were from sepsis, one from hypothermia, one from diverticular perforation and one patient elected to stop all medications.

**Table 5:** Diagnosis and age of patients who died during admission (*n* = 15)

Patient	Age at death	Diagnosis on admission	Primary ICD-10	Secondary ICD-10
1	60	Hypothermia	XIX Injury, poisoning and certain other consequences of external causes	N/A
2	66	Renal failure	XIV Diseases of the genitourinary system	N/A
3	56	Distal tibial fracture	XIX Injury, poisoning and certain other consequences of external causes	XX External causes of morbidity and mortality
4	45	Renal failure	XIV Diseases of the genitourinary system	N/A
5	64	Biliary sepsis	I Certain infectious and parasitic diseases	II Neoplasms
6	54	Road traffic collision, cardiac arrest	XX External causes of morbidity and mortality	XIX Injury, poisoning and certain other consequences of external causes
7	29	Renal amyloidosis	XIV Diseases of the genitourinary system	N/A
8	71	Fall, cognitive impairment	XVIII Symptoms, signs and abnormal clinical and laboratory findings, not elsewhere classified	VI Diseases of the nervous system
9	39	Cervical cancer	II Neoplasms	N/A
10	55	Passive suicide – stopped all medications	V Mental and behavioural disorders	XX External causes of morbidity and mortality
11	61	Road traffic collision – hit by lorry	XX External causes of morbidity and mortality	XIX Injury, poisoning and certain other consequences of external causes
12	68	Metastatic lung cancer	II Neoplasms	XXI Factors influencing health status and contact with health services
13	77	Sepsis	I Certain infectious and parasitic diseases	N/A
14	60	Renal cancer	II Neoplasms	XIV Diseases of the genitourinary system
15	48	Diverticular perforation	XI Diseases of the digestive system	N/A

### Admission and discharge characteristics

#### Trauma related admission

Of all admissions, 17.1% (*n* = 194) documented trauma as contributing to the admission (Table 6). The most common trauma was overdose (6.3%, *n* = 72), followed by accidents (4.6%, *n* = 52), assault (3.2%, *n* = 36), road traffic collision (RTC) (1.9%, *n* = 21) and documented suicide attempt (1.1% *n* = 13%). Some records (7%, *n* = 82) were missing this information.

**Table 6:** Characteristics of analysed admissions

Characteristic	Total patients (*n*)	Percent
Self-discharge	49	4.3
Type of encounter		
Planned	30	2.6
Unplanned	1105	97.4
Surgery or procedure documented		
Surgery	56	4.9
Procedure	22	1.9
N/A	997	87.8
Missing data	60	5.3
Admission associated with trauma		
Accident RTC	21	1.9
Accident other	52	4.6
Assault	36	3.2
Overdose	74	6.5
Suicide attempt	13	1.1
N/A	857	75.5
Missing data	82	7.2
Housing status on discharge		
Improved	266	23.4
Same	418	36.8
Worse	18	1.6
Died	15	1.3
Unknown	418	36.8

#### Drug and alcohol use

**Fig. 4:**
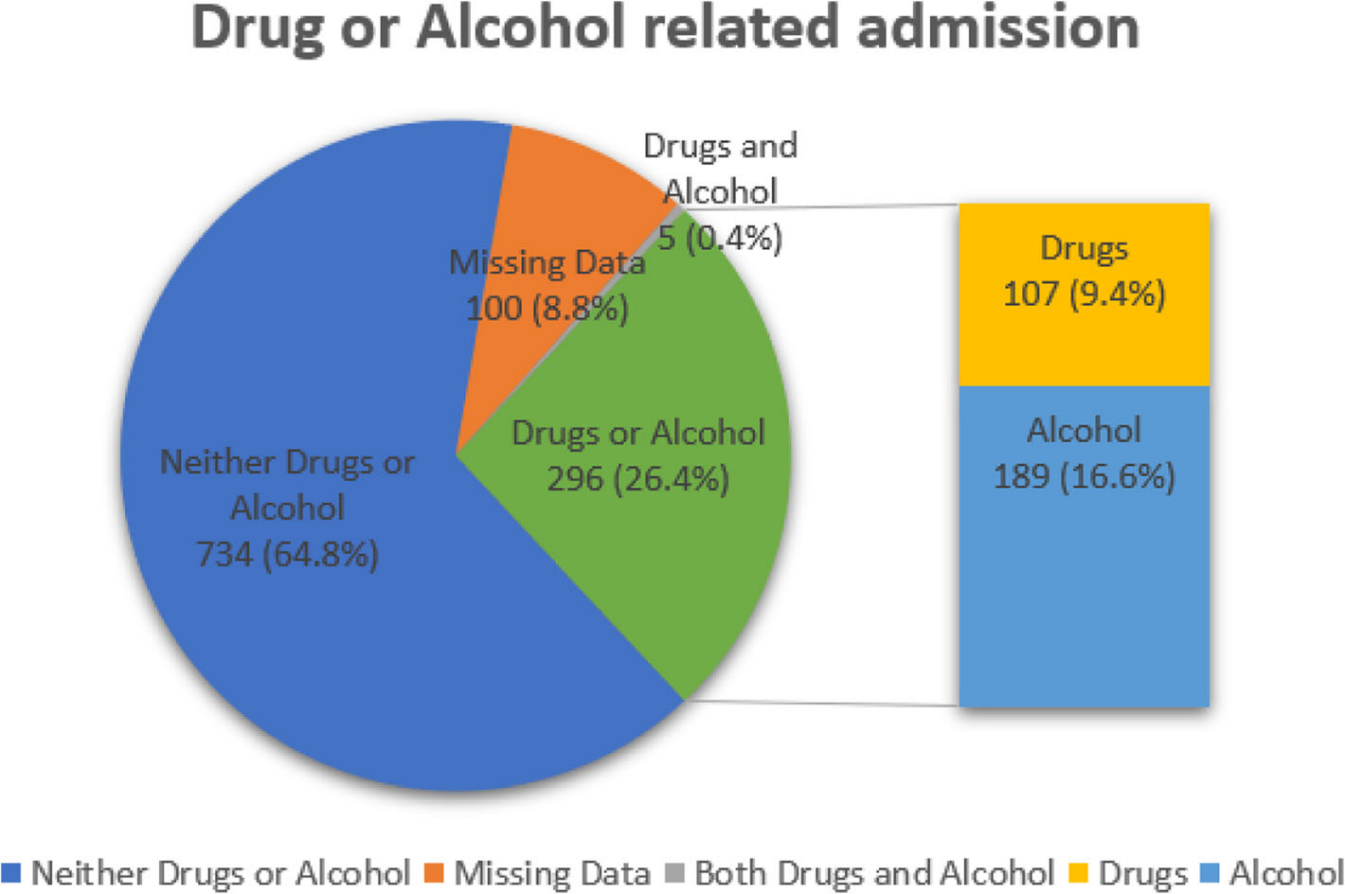
Drug or alcohol related admission (both, nether and missing data represented)

More than a quarter of admissions (26.4%, *n* = 300) were related to the immediate consequences of drug or alcohol use (e.g. acute alcohol withdrawal, seizures, or intoxication related falls) (Fig. 4). Alcohol was the main contributor affecting one fifth of admissions (20%, *n* = 193) with drugs contributing to 9.8% (*n* = 112). Five (0.4%) of these admissions involved both drugs and alcohol.

#### Housing outcome on discharge

Nearly two thirds (*n* = 684, 60.2%) of patients’ accommodation status remained the same or improved on discharge, with 23.4% improving (*n* = 266) and a small number of patient’s (*n* = 18, 1.6%) housing status deteriorated). For a large proportion of participants (*n* = 418, 36.8%) a discharge destination was not reported in the notes and 15 patients died (1.3%) during admission.

#### Secondary care usage

Across the sample there were 767 A&E attendances in the 120-days prior to admission averaging 0.68 per patient, decreasing to 0.65 (735 A&E attendances) during the 120-days after discharge (*p* = 0.31) (Table 7). There were 135 planned admissions (average 0.12 admissions) in the 120-days prior to the index admission, this increased to 283 planned admissions (average 0.25 admissions) in the 120-days following discharge (*p* = 0.03). The 610 unplanned admissions (average 0.54 admissions) in the 120-days prior to admission increased to 654 unplanned admissions (average 0.58 admissions) following discharge (*p* = 0.12). The average length of stay for both planned and unplanned admissions 120-days before admission was 3.49 which increased slightly to 3.9 by 120-days post-discharge (*p* = 0.15).

**Table 7:** Secondary care usage 120-days prior to admission and 120-days following discharge

Characteristic		Average (SD)	Total	Maximum attendances
Index admission	Average LOS (days)	14	14	
Number of secondary care attendances 120-days prior to admission	A&E	0.68 (SD 2.36)	767	63
	Planned admission	0.12 (SD 0.06)	135	48
	Unplanned admission	0.54 (SD 0.03)	610	9
	Total bed days	3.49 (0.33)	3965	187
	Average LOS (days)		5	
Number of secondary care attendances 120-days following discharge	A&E	0.65 (SD 2.11)	735	32
	Planned admission	0.25 (SD 2.79)	283	51
	Unplanned admission	0.58 (SD 1.31)	654	12
	Total bed days	3.90 (SD 11.0)	4430	101
	Average LOS (days)		5	

Most patients did not use secondary care before or after the index admission, so the median and mode for A&E attendances and secondary care usage pre and post-index admission was 0. The maximum A&E attendances by one patient prior to index admission was 63, which reduced to 32 following Pathway contact. The most unplanned admissions increased from 48 prior to index admission, to 51. The most unplanned admissions by a patient were 9 prior to index admission, which increased to 12 following assessment. One patient totaled 187 bed days prior to index admission, following this, the longest total bed days were 101 days. This reflects complex health needs which are likely to need further periods of hospital care.

## Discussion

### Statement of principal findings

This retrospective analysis outlines the characteristics of hospital admissions for people experiencing homelessness in seven specialist hospital-based homelessness teams in the UK. The most commonly recorded diagnoses at admission were categorised as mental and behavioural disorders (alcohol intoxication or withdrawal, self-harm, overdose, suicidal ideation or depression), external causes of morbidity and mortality (assault, road traffic collisions, trauma) and injury, poisoning and other consequences of external causes (poisoning, head injuries and fractures). Most admissions had a physical component and physical illness was commonly associated with substance misuse or mental illness.

Within this study, unplanned secondary care usage was not consistently reduced following support and intervention from a hospital Pathway team. Slight reductions in A&E attendances and a doubling of planned hospital care following contact with a homeless team were observed. Patients experiencing homelessness frequently disengage with healthcare and most deaths in this population are due to treatable medical conditions. Therefore, a significant increase in attending planned admissions is a positive finding as it suggests that patients are receiving necessary investigations, such as follow up endoscopies, procedures, and treatment such as surgery or cancer therapy. Any health attendance is an opportunity to review health, address wider needs, and engage homeless patients into services.

Most patients maintained or improved their housing status on discharge. However, a challenge that was faced within the data was the inconsistent reporting of discharge status. Where data was available, over 60% of patients (*n* = 684), were discharged to the same type of accommodation or a more appropriate discharge destination. The value in maintaining or improving housing status observed in this research cannot be underestimated, and a great deal of advocacy and negotiation of additional support may be needed to ensure that a patient can return to a hostel that was struggling to support them. The accommodation status of nearly 80% of Bradford patients improved following admissions, due largely to patients being discharged to Bradford Respite Intermediate Care Support Service (BRICSS), a home from hospital destination for patients experiencing homelessness or with unsuitable accommodation on discharge [40]. More provision of step-down intermediate care (medical respite) could further improve outcomes for homeless patients [41].

### Links to existing literature

Observational studies have found reduced admitted bed days following the introduction of a Pathway team, compared to data for similar patients before the intervention, or matched patients not offered the intervention [10, 33, 34, 42]. A randomised controlled trial of a Pathway team intervention did not show reduced total admitted bed days after the intervention, but did show cost-effectiveness and improved health outcomes [35].

The average age of people experiencing homelessness admitted to hospital in this research was 43 years. This is in-line with previous research from Dublin in which the use of unscheduled emergency department and inpatient care between the housed and homeless population was compared. In Dublin, the average age of inpatients experiencing homelessness was 44 years, whereas that of housed patients was 61 years [43].

Within this study, the average length of stay for admitted patients experiencing homelessness was 14-days. This is consistent with other evidence [3]. For the general population, average length of inpatient stay is 6 days [44]. This difference is explained by the need to resolve multiple health problems alongside wider needs to deliver a safe discharge and community support. This is nearly three times the average of 5 days duration of admission for patients in the 120-days before and after the index admission. This suggests that Pathway teams are being appropriately involved at times of complex admissions. The Pathway model is a complex intervention.

A systematic review and meta-analysis of morbidity and mortality in homeless individuals (amongst others) found substantial indices of (I) Infectious diseases and (V) mental and behavioural disorders, a finding that was similar in our research [2]. Previous research exploring the epidemiology of homeless people in high-income countries, including the UK, reports that 9% of admissions to hospital for this group were prompted by unintentional injuries [21]*.* Our figures were slightly higher with 12.4% admitted for (XIX) Injury, poisoning and certain other consequences of external causes (hypothermia, poisoning, head injury, stabbed, fall) and 18.7% for XX External causes of morbidity and mortality (RTC, assault, head trauma, dog bite). Fazel concludes that substance misuse and mental health disorders are risk factors for high secondary care usage, an observation consistent with our analysis of bi- and tri-morbidity [21].

In this research, cause of mortality data was limited to those who died whilst an inpatient within the study period (*n =* 15). The causes of death reported (XX External causes of morbidity and XIX Injury, poisoning and certain other consequences) were similar to those reported in an analysis of 130 homeless deaths in East London [45]. The main morbidities and causes of mortality reported in this study are consistent with the life experiences of people living with poverty, deprivation and social exclusion [2]. Data from the office of national statistics [24] and evidence from Glasgow [46] also demonstrates how premature morbidity and mortality in deprived populations is frequently due to substance use disorders, suicide and violence. In response to the paucity of data around causes, and number of deaths among people experiencing homelessness in the UK, the Bureau of Investigative Journalism have launched an enquiry and are collating data in this area [47]*.*

This retrospective evaluation focused on admissions and secondary health care usage for people identified as experiencing homelessness. While exploring which interventions are effective in supporting this group was not our focus, a recent Lancet systematic review, Luchenski et al. explored “what works” in inclusion health [1]. They reported that providing housing and engaging homeless persons with mental health services (Housing First model) decreased health service use. This correlates with our hypothesis of maintaining housing outcomes for this cohort. Respite care can also reduce secondary care usage, a process supported by Hwang and Burns [48].

### Strengths and limitations of the study

#### Identifying participants

Homelessness is not routinely coded in NHS data. Most studies exploring inpatients experiencing homelessness are therefore dependent on whether the hospital system has coded the patient as having “no fixed abode” (NFA) or no address [49]. Many hospitals will reproduce an old address automatically meaning patients are not correctly identified as potentially being homeless. This analysis drew on the records of specialist homelessness teams, as such, each patient was individually identified as being homeless. This included case notes and discharge summaries which facilitated a more robust understanding of each inpatient episode than would have been obtained by relying on computer coding. Pathway teams are embedded within hospitals meaning that individuals who are or are at risk of becoming homeless are often referred to them. Despite this, it is possible that not all homeless patients presenting at included hospitals were referred to Pathway teams.

#### Inconsistencies and limitations of included data

Limited resources and a lack of consistent information in records and discharge summaries posed challenges. The process of collecting and analysing data was labour intensive. One researcher (HF) manually reviewed paper files and collated information held in various locations across the country. Data was often missing or inconsistently recorded, meaning that estimates provided may represent underestimates.

Trends in healthcare access and data recording processes presented issues. Many patients experiencing homelessness may have multiple patient records (for several reasons including different name spellings, and varied date of birth), attendances at other hospitals were not collected. There were a small proportion of duplicate patients who were re-referred and assessed within the six-month period, which was not unexpected given the patterns of healthcare usage of homeless patients described elsewhere [50].

Within 12-months of discharge, 50 patients (5%) from the cohort died. There is often a delay before deaths in the community are updated on hospital records and this analysis did not cross reference other sources of information. It is possible therefore that other deaths occurred but were not recorded or included in this analysis.

#### Measuring the impact of hospital-based interventions

In times of austerity any new health intervention is usually required to offer savings, for example by reducing delayed discharge, or preventing re-admissions. Unplanned hospital admission marks a threshold in deteriorating health, increasing the probability of further acute admissions. Achieving sustained improvements in the health of homeless people needs investment in a range of community health, housing, care and support services [51].

Although total A&E number of attendances was reduced following the index admission with referral and assessment by a Pathway team, unplanned admissions and bed days increased. As these are complex presentations requiring acute care and a Pathway intervention, it seems likely that the patient is on a downward trajectory which would result in more secondary care usage. This theory is supported by the recent report by Waugh et al. on health and homelessness in Scotland [52]. Waugh described healthcare activity before and after the first homelessness assessment which concludes that this threshold marks a deterioration in health and that homelessness as an event increases secondary care usage [52]. However, we also found a statistically significant increase in planned secondary care usage (*p* = 0.03) following the index admission, suggesting better engagement and more appropriate care.

Our data shows that, when dealing with complex patients who have passed the threshold of requiring acute admission, “before and after data” without a control group is not an appropriate method of measuring the effectiveness of the intervention.

## Conclusions

### Meaning of the study: possible explanations and implications for clinicians and policymakers

Across the UK and worldwide, homeless people are being admitted to hospital with acute medical conditions frequently associated with substance use disorders and mental illness [43]. Pathway teams are effective at care coordination and improving wider outcomes including housing, care and support at discharge. The need for these services is underpinned by ethical practice and parity of care to prevent inequity. Rather than seeking in-year savings from the care of deprived and complex patients we should instead apply Marmot’s principle of “proportionate universality” [14] and increase resources to meet the needs of some of society’s most vulnerable people [15, 19]. Specialist inclusion health services will continue to be needed but are being challenged by the increasing volume and complexity of patients seen, a lack of appropriate and affordable housing and suitable places of care, a hostile environment towards migrants, overwhelmed social care systems, lack of support services and reducing provision of drug and alcohol services. Long term, government and policy makers must take three actions. Firstly, redress the chronic underfunding of all public services and local authorities alongside mandatory data collection of housing status and recording of deaths of homeless people. Secondly, mandate specialist support (such as Pathway teams) with education and training of all staff to increase the knowledge and skills to address the needs of inclusion health groups and those living in poverty. Thirdly, take steps to end homelessness through the provision of a range of social housing and places of care with adequate welfare provision to cover the costs.

### Unanswered questions and future research

Consideration needs to be given to the types of outcome used to assess whether an intervention for people experiencing homelessness with health needs adds value, rather than simply being cost saving. While this retrospective review provides insights into the reasons for admission, morbidity, mortality and secondary health care usage, a more robust design including matched case controls [42] would enable firmer conclusions to be drawn regarding the impact of the Pathway teams’ intervention.

Traditionally used outcome measures, such as reductions in health care usage may not accurately reflect positive outcomes for this group. Further research is needed to assess the impact of improved community support on admissions that may be considered an “adversity related admission” such as self-harm, drug misuse, alcohol misuse or violence.

## Supplementary information

**Additional file 1.** Data extraction template. Blank data extraction document for recording data extracted per patient.

**Additional file 2.** Full diagnoses of each admitted patient with ICD-10 categories. Full break-down of diagnoses within each ICD-10 category with numbers of patients diagnosed.

## Data Availability

The datasets used and/or analysed during the current study are available from the corresponding author on reasonable request.

## Abbreviations

A&E: Accident and Emergency
GP: General practitioner
ICD-10: International Classification of Diseases, 10th Revision
IH: Inclusion health
IHG: Inclusion health groups
MCCD: Medical certificate for cause of death
NFA: No fixed abode
NRPF: No recourse to public funds
OT: Occupational therapist
RTC: Road traffic collision
SMR: Standardised mortality rates
UK: United Kingdom
