# Supplementary material for: Secondary care usage and characteristics of hospital inpatients referred to a UK homeless health team: a retrospective service evaluation
Source: BMC Health Serv Res. 2019 Nov 21;19:857. doi: 10.1186/s12913-019-4620-1 (PMC6868755; doi:10.1186/s12913-019-4620-1)
Supplement: Supplementary file 2 — Additional file 2. Full diagnoses of each admitted patient with ICD-10 categories. Full break-down of diagnoses within each ICD-10 category with numbers of patients diagnosed. [file 12913_2019_4620_MOESM2_ESM.docx]

| **ICD-10 code** | ***Diagnosis*** | *Primary diagnosis*  *= n* | *Primary diagnosis = n (%)* | *Secondary diagnosis*  *= n* | *Secondary diagnosis*  *= n (%)* | *Total prevalence = n* |
| --- | --- | --- | --- | --- | --- | --- |
| I Certain infectious and parasitic diseases | *Bacteraemia* | 3 | 54 (4.8) | 3 | 16 (1.4) | 70 (6.2) |
|  | *Hepatitis C* | 3 |  | 3 |  |  |
|  | *Human Immunodeficiency Virus (HIV)* | 14 |  | 3 |  |  |
|  | *Meningitis* | 2 |  | 0 |  |  |
|  | *Sepsis* | 15 |  | 6 |  |  |
|  | *Toxoplasmosis* | 1 |  | 0 |  |  |
|  | *Tuberculosis (TB)* | 15 |  | 1 |  |  |
|  | *Viral illness* | 1 |  | 0 |  |  |
| II Neoplasms | *Acute myeloid leukaemia (AML)* | 2 | 30 (2.6) | 1 | 8 (0.7) | 38 (3.3) |
|  | *Bladder cancer* | 0 |  | 1 |  |  |
|  | *Brain tumour* | 1 |  | 0 |  |  |
|  | *Breast cancer* | 1 |  | 1 |  |  |
|  | *Cancer unspecified* | 6 |  | 0 |  |  |
|  | *Cervical cancer* | 1 |  | 0 |  |  |
|  | *Gastrointestinal stromal tumour (GIST)* | 2 |  | 0 |  |  |
|  | *Hepatocellular cancer* | 1 |  | 0 |  |  |
|  | *Laryngeal cancer* | 2 |  | 1 |  |  |
|  | *Lung cancer* | 7 |  | 0 |  |  |
|  | *Multiple myeloma* | 2 |  | 2 |  |  |
|  | *Oesophageal cancer* | 1 |  | 0 |  |  |
|  | *Pancreatic cancer* | 0 |  | 1 |  |  |
|  | *Pituitary cancer* | 1 |  | 0 |  |  |
|  | *Prostate cancer* | 0 |  | 1 |  |  |
|  | *Rectal cancer* | 1 |  | 0 |  |  |
|  | *Renal cell cancer* | 2 |  | 0 |  |  |
| III Diseases of the blood and blood-forming organs and certain disorders involving the immune mechanism | *Anaemia* | 2 | 12 (1.1) | 2 | 5 (0.4) | 17 (1.5) |
|  | *Myelodysplastic syndrome* | 1 |  | 0 |  |  |
|  | *Neutropenic sepsis* | 0 |  | 2 |  |  |
|  | *Pancytopenia* | 1 |  | 1 |  |  |
|  | *Sickle cell disease* | 1 |  | 0 |  |  |
|  | *Vaso-occlusive crisis* | 7 |  | 0 |  |  |
| IV Endocrine, nutritional and metabolic diseases | *Alcohol withdrawal* | 0 | 39 (3.4) | 32 | 45 (4.0) | 84 (7.4) |
|  | *Diabetic foot* | 4 |  | 0 |  |  |
|  | *Diabetic ketoacidosis* | 14 |  | 0 |  |  |
|  | *Hyperglycaemia* | 10 |  | 3 |  |  |
|  | *Hyperglycaemic hyperosmolar state* | 4 |  | 0 |  |  |
|  | *Hypocalcaemia* | 1 |  | 0 |  |  |
|  | *Hypoglycaemia* | 1 |  | 0 |  |  |
|  | *Hypokalaemia* | 2 |  | 0 |  |  |
|  | *Hyponatraemia* | 0 |  | 1 |  |  |
|  | *Korsakoffs psychosis* | 0 |  | 4 |  |  |
|  | *Re-feeding syndrome* | 1 |  | 1 |  |  |
|  | *Syndrome of inappropriate antidiuretic hormone secretion (SIADH)* | 1 |  | 0 |  |  |
|  | *Thyroid mass* | 1 |  | 0 |  |  |
|  | *Wernicke’s encephalopathy* | 0 |  | 4 |  |  |
| V Mental and behavioural disorders | Addiction | 5 | 210 (18.5) | 22 | 111 (9.8) | 321 (28.3) |
|  | *Alcohol* | 0 |  | 27 |  |  |
|  | *Alcohol induced seizure* | 0 |  | 30 |  |  |
|  | *Alcohol intoxication* | 38 |  | 11 |  |  |
|  | *Alcohol poisoning* | 7 |  | 1 |  |  |
|  | *Alcohol withdrawal* | 36 |  | 6 |  |  |
|  | *Bipolar affective disorder* | 1 |  | 0 |  |  |
|  | *Deliberate self-harm* | 5 |  | 0 |  |  |
|  | *Depression* | 3 |  | 3 |  |  |
|  | *Hallucinations* | 2 |  | 1 |  |  |
|  | *Learning difficulties* | 1 |  | 0 |  |  |
|  | *Mental illness* | 5 |  | 2 |  |  |
|  | *Overdose* | 74 |  | 4 |  |  |
|  | *Panic attack* | 1 |  | 0 |  |  |
|  | *Pseudo-seizure* | 1 |  | 0 |  |  |
|  | *Psychosis* | 5 |  | 0 |  |  |
|  | *Schizophrenia* | 1 |  | 0 |  |  |
|  | *Suicidal ideation* | 18 |  | 3 |  |  |
|  | *Suicide attempt* | 7 |  | 1 |  |  |
| VI Diseases of the nervous system | *Cognitive impairment* | 0 | 74 (6.5) | 1 | 5 (0.4) | 79 (7.0) |
|  | *Encephalitis* | 3 |  | 1 |  |  |
|  | *Focal neurology* | 1 |  | 0 |  |  |
|  | *Headache* | 3 |  | 0 |  |  |
|  | *Intracranial hypertension* | 1 |  | 0 |  |  |
|  | *Miller-fisher* | 1 |  | 0 |  |  |
|  | *Motility disorder* | 1 |  | 0 |  |  |
|  | *Multiple sclerosis* | 2 |  | 0 |  |  |
|  | *Peripheral neuropathy* | 0 |  | 1 |  |  |
|  | *Seizure* | 53 |  | 2 |  |  |
|  | *Spinal cord compression* | 1 |  | 0 |  |  |
|  | *Status epilepticus* | 7 |  | 0 |  |  |
|  | *Transverse myelitis* | 1 |  | 0 |  |  |
| VII Diseases of the eye and adnexa | *Blindness, cataracts, glaucoma, visual disturbance,* | 0 | 0 (0) | 0 | 0 (0) | 0 (0) |
| VIII Diseases of the ear and mastoid process | *Otitis externa, otitis media, Meniere’s disease, perforated ear drum* | 0 | 0 (0) | 0 | 0 (0) | 0 (0) |
| IX Diseases of the circulatory system | *Abdominal aortic aneurysm (AAA)* | 0 | 81 (7.1) | 1 | 20 (1.8) | 101 (8.9) |
|  | *Acute limb ischaemia* | 4 |  | 0 |  |  |
|  | *Aneurysm* | 3 |  | 0 |  |  |
|  | *Angina* | 6 |  | 0 |  |  |
|  | *Aortic dissection* | 1 |  | 0 |  |  |
|  | *Arteriovenous fistula* | 0 |  | 2 |  |  |
|  | *Atrial fibrillation (AF)* | 0 |  | 1 |  |  |
|  | *Atrial flutter* | 1 |  | 0 |  |  |
|  | *Cardiomyopathy* | 1 |  | 0 |  |  |
|  | *Chronic venous insufficiency* | 1 |  | 0 |  |  |
|  | *Deep vein thrombosis (DVT)* | 15 |  | 7 |  |  |
|  | *Heart failure* | 5 |  | 1 |  |  |
|  | *Infective endocarditis* | 2 |  | 0 |  |  |
|  | *Ischaemic heart disease* | 1 |  | 0 |  |  |
|  | *Leg ulcers* | 10 |  | 3 |  |  |
|  | *Malignant hypertension* | 3 |  | 0 |  |  |
|  | *Myocardial infarction (MI)* | 4 |  | 0 |  |  |
|  | *Oesophageal varices* | 4 |  | 1 |  |  |
|  | *Palpitations* | 1 |  | 0 |  |  |
|  | *Pericardial effusion* | 2 |  | 0 |  |  |
|  | *Peripheral artery disease* | 2 |  | 0 |  |  |
|  | *Post-thrombotic syndrome* | 1 |  | 1 |  |  |
|  | *Pulmonary embolism (PE)* | 4 |  | 1 |  |  |
|  | *Pulseless electrical activity (PEA)* | 0 |  | 2 |  |  |
|  | *Septic emboli* | 2 |  | 0 |  |  |
|  | *Stroke* | 6 |  | 0 |  |  |
|  | *Valve repair* | 1 |  | 0 |  |  |
|  | *Ventricular tachycardia (VT)* | 1 |  | 0 |  |  |
| X Diseases of the respiratory system | *Aspergilloma* | 1 | 83 (7.3) | 0 | 35 (3.1) | 118 (10.4) |
|  | *Aspiration pneumonia* | 2 |  | 5 |  |  |
|  | *Asthma* | 3 |  | 0 |  |  |
|  | *Bronchitis* | 2 |  | 0 |  |  |
|  | *Community acquired pneumonia (CAP)* | 32 |  | 19 |  |  |
|  | *Empyema* | 5 |  | 0 |  |  |
|  | *Haemothorax* | 0 |  | 1 |  |  |
|  | *Healthcare acquired pneumonia (HAP)* | 1 |  | 2 |  |  |
|  | *Infective exacerbation of COPD* | 16 |  | 1 |  |  |
|  | *Lower respiratory tract infection* | 8 |  | 2 |  |  |
|  | *Lung abscess* | 0 |  | 1 |  |  |
|  | *Lung lesion* | 0 |  | 1 |  |  |
|  | *Pleural effusion* | 2 |  | 0 |  |  |
|  | *Pneumocystis pneumonia (PCP)* | 1 |  | 0 |  |  |
|  | *Pneumothorax* | 1 |  | 1 |  |  |
|  | *Pulmonary fibrosis* | 1 |  | 0 |  |  |
|  | *Smoke inhalation* | 1 |  | 2 |  |  |
|  | *Type 2 respiratory failure* | 7 |  | 0 |  |  |
| XI Diseases of the digestive system | *Abscess* | 1 | 92 (8.1) | 0 | 14 (1.2) | 106 (9.3) |
|  | *Ascites* | 3 |  | 0 |  |  |
|  | *Cholecystitis* | 2 |  | 0 |  |  |
|  | *Cirrhosis of liver* | 3 |  | 0 |  |  |
|  | *Colon oedema* | 1 |  | 0 |  |  |
|  | *Colonoscopy* | 1 |  | 0 |  |  |
|  | *Constipation* | 1 |  | 0 |  |  |
|  | *Crohn’s disease* | 3 |  | 0 |  |  |
|  | *Decompensated alcoholic liver disease* | 10 |  | 3 |  |  |
|  | *Diarrhoea* | 0 |  | 1 |  |  |
|  | *Diverticulitis* | 3 |  | 0 |  |  |
|  | *Fistula* | 1 |  | 0 |  |  |
|  | *Gall bladder* | 1 |  | 0 |  |  |
|  | *Gastritis* | 8 |  | 0 |  |  |
|  | *Gastroenteritis* | 5 |  | 1 |  |  |
|  | *Gastrointestinal bleed* | 6 |  | 3 |  |  |
|  | *Hemicolectomy* | 1 |  | 0 |  |  |
|  | *Hepatitis* | 3 |  | 1 |  |  |
|  | *Hernia* | 2 |  | 1 |  |  |
|  | *Necrotic pancreas* | 1 |  | 0 |  |  |
|  | *Oesophageal stricture* | 4 |  | 0 |  |  |
|  | *Oesophagitis* | 4 |  | 0 |  |  |
|  | *Pancreatitis* | 15 |  | 2 |  |  |
|  | *Peptic ulcer* | 3 |  | 0 |  |  |
|  | *Perforation* | 5 |  | 1 |  |  |
|  | *Rectal prolapse* | 3 |  | 0 |  |  |
|  | *Small bowel obstruction (SBO)* | 0 |  | 1 |  |  |
|  | *Ulcerative colitis* | 1 |  | 0 |  |  |
|  | *Volvulus* | 1 |  | 0 |  |  |
| XII Diseases of the skin and subcutaneous tissue | *Abscess* | 33 | 78 (6.9) | 5 | 15 (1.3) | 93 (8.2) |
|  | *Cellulitis* | 30 |  | 8 |  |  |
|  | *Cyst excision* | 1 |  | 0 |  |  |
|  | *Dermatitis* | 2 |  | 0 |  |  |
|  | *Infected prosthesis* | 1 |  | 0 |  |  |
|  | *Leg ulcers* | 6 |  | 2 |  |  |
|  | *Necrosis* | 1 |  | 0 |  |  |
|  | *Pilonidal fistula* | 1 |  | 0 |  |  |
|  | *Psoriasis* | 1 |  | 0 |  |  |
|  | *Vasculitis* | 2 |  | 0 |  |  |
| XIII Diseases of the musculoskeletal system and connective tissue | *Arthritis* | 1 | 45 (4.0) | 1 | 15 (1.3) | 60 (5.3) |
|  | *Back pain* | 4 |  | 2 |  |  |
|  | *Cervical myelopathy* | 1 |  | 0 |  |  |
|  | *Cut off hand* | 1 |  | 0 |  |  |
|  | *Discitis* | 1 |  | 1 |  |  |
|  | *Dislocation* | 2 |  | 0 |  |  |
|  | *Joint replacement* | 0 |  | 1 |  |  |
|  | *Limb infection* | 2 |  | 1 |  |  |
|  | *Limb pain* | 5 |  | 3 |  |  |
|  | *Limb swelling* | 7 |  | 0 |  |  |
|  | *Necrotising fasciitis* | 1 |  | 0 |  |  |
|  | *Olecranon bursitis* | 2 |  | 0 |  |  |
|  | *Osteoarthritis* | 1 |  | 0 |  |  |
|  | *Osteomyelitis* | 4 |  | 2 |  |  |
|  | *Polyarthritis* | 2 |  | 0 |  |  |
|  | *Psoas abscess* | 1 |  | 0 |  |  |
|  | *Rectus sheath haematoma* | 0 |  | 1 |  |  |
|  | *Rhabdomyolysis* | 1 |  | 0 |  |  |
|  | *Septic arthritis* | 7 |  | 2 |  |  |
|  | *Soft tissue injury* | 1 |  | 1 |  |  |
|  | *Systemic lupus erythematosus (SLE)* | 1 |  | 0 |  |  |
| XIV Diseases of the genitourinary system | *Acute kidney injury (AKI)* | 2 | 37 (3.3) | 3 | 8 (0.7) | 45 (4.0) |
|  | *Chronic kidney disease (CKD)* | 4 |  | 0 |  |  |
|  | *Cystitis* | 1 |  | 0 |  |  |
|  | *Gynaecology problem* | 1 |  | 0 |  |  |
|  | *Pelvic inflammatory disease (PID)* | 1 |  | 0 |  |  |
|  | *Pyelonephritis* | 2 |  | 0 |  |  |
|  | *Renal amyloidosis* | 1 |  | 0 |  |  |
|  | *Renal failure* | 7 |  | 1 |  |  |
|  | *Renal mass* | 0 |  | 1 |  |  |
|  | *Renal stone* | 2 |  | 0 |  |  |
|  | *Renal transplant* | 2 |  | 0 |  |  |
|  | *Tubo-ovarian abscess* | 1 |  | 0 |  |  |
|  | *Tubular nephritis* | 1 |  | 0 |  |  |
|  | *Uraemia* | 1 |  | 0 |  |  |
|  | *Urethral stricture* | 2 |  | 0 |  |  |
|  | *Urinary tract infection (UTI)* | 5 |  | 3 |  |  |
|  | *Urosepsis* | 4 |  | 0 |  |  |
| XV Pregnancy, childbirth and the puerperium | *Caesarean* | 5 | 9 (0.8) | 0 | 0 (0) | 9 (0.8) |
|  | *Disorders of pregnancy* | 1 |  | 0 |  |  |
|  | *Labour* | 1 |  | 0 |  |  |
|  | *Miscarriage* | 0 |  | 0 |  |  |
|  | *Multiple gestation* | 0 |  | 0 |  |  |
|  | *Obstetric death* | 0 |  | 0 |  |  |
|  | *Postpartum haemorrhage (PPH)* | 1 |  | 0 |  |  |
|  | *Termination* | 1 |  | 0 |  |  |
| XVI Certain conditions originating in the perinatal period | *Birth trauma, newborn disorders, perinatal infections,* | 0 | 0 (0) | 0 | 0 (0) | 0 (0) |
|  |  |  |  |  |  |  |
| XVII Congenital malformations, deformations and chromosomal abnormalities | *Arteriovenal malformation (AVM)* | 0 | 0 (0) | 1 | 1 (0.1) | 1 (0.1) |
|  | *Atrial septal defect, microcephaly, spina bifida, transposition of the great vessels* | 0 |  | 0 |  |  |
|  |  |  |  |  |  |  |
| XVIII Symptoms, signs and abnormal clinical and laboratory findings, not elsewhere classified | *Abdominal pain* | 9 | 84 (7.4) | 3 | 29 (2.6) | 113 (10.0) |
|  | *Acute confusion* | 7 |  | 3 |  |  |
|  | *Chest pain* | 7 |  | 0 |  |  |
|  | *Collapse* | 16 |  | 6 |  |  |
|  | *Diarrhoea* | 1 |  | 0 |  |  |
|  | *Dysphagia* | 1 |  | 1 |  |  |
|  | *Epistaxis* | 2 |  | 0 |  |  |
|  | *Fatigue* | 0 |  | 1 |  |  |
|  | *Loss of consciousness* | 6 |  | 1 |  |  |
|  | *Pain* | 3 |  | 2 |  |  |
|  | *Shock* | 0 |  | 1 |  |  |
|  | *Shortness of breath* | 2 |  | 4 |  |  |
|  | *Sore throat* | 1 |  | 0 |  |  |
|  | *Splenomegaly* | 0 |  | 1 |  |  |
|  | *Syncope* | 5 |  | 0 |  |  |
|  | *Unwell* | 16 |  | 2 |  |  |
|  | *Vomiting* | 8 |  | 4 |  |  |
| XIX Injury, poisoning and certain other consequences of external causes | *Blood loss* | 1 | 61 (5.4) | 1 | 80 (7.0) | 141 (12.4) |
|  | *Burns* | 0 |  | 1 |  |  |
|  | *Collection* | 1 |  | 0 |  |  |
|  | *Compartment syndrome* | 0 |  | 2 |  |  |
|  | *Dislocation* | 0 |  | 2 |  |  |
|  | *Fracture* | 31 |  | 33 |  |  |
|  | *Head Injury* | 10 |  | 9 |  |  |
|  | *Hypothermia* | 2 |  | 0 |  |  |
|  | *Injury unspecified* | 0 |  | 7 |  |  |
|  | *Laceration* | 1 |  | 12 |  |  |
|  | *Meniscal tear* | 1 |  | 0 |  |  |
|  | *Numbness* | 1 |  | 0 |  |  |
|  | *Poisoning* | 3 |  | 0 |  |  |
|  | *Subdural haemorrhage/brain injury* | 9 |  | 12 |  |  |
|  | *Surgical infection* | 1 |  | 1 |  |  |
| XX External causes of morbidity and mortality | *Accident* | 2 | 89 (7.8) | 25 | 123 (10.8) | 212 (18.7) |
|  | *Assault* | 26 |  | 0 |  |  |
|  | *Deliberate self-harm* | 5 |  | 9 |  |  |
|  | *Dog bite* | 1 |  | 0 |  |  |
|  | *Fall* | 11 |  | 5 |  |  |
|  | *Fire* | 2 |  | 0 |  |  |
|  | *Overdose* | 0 |  | 73 |  |  |
|  | *Road traffic collision* | 21 |  | 0 |  |  |
|  | *Stabbed* | 17 |  | 0 |  |  |
|  | *Trauma* | 4 |  | 11 |  |  |
| XXI Factors influencing health status and contact with health services | *Abortion* | 0 | 20 (1.8) | 1 | 57 (5.0) | 77 (6.8) |
|  | *Amputation* | 1 |  | 5 |  |  |
|  | *Angiography* | 0 |  | 2 |  |  |
|  | *Chemotherapy* | 0 |  | 5 |  |  |
|  | *Colonoscopy* | 0 |  | 1 |  |  |
|  | *Coronary artery bypass graft (CABG)* | 1 |  | 2 |  |  |
|  | *Dialysis* | 0 |  | 4 |  |  |
|  | *Double cord transplant* | 1 |  | 0 |  |  |
|  | *Refused intervention* | 0 |  | 2 |  |  |
|  | *Skin graft replacement* | 1 |  | 0 |  |  |
|  | *Social* | 0 |  | 2 |  |  |
|  | *Stent insertion* | 1 |  | 3 |  |  |
|  | *Surgery* | 15 |  | 30 |  |  |
| XXII Codes for special purposes | *Social concerns* | 1 | 1 (0.1) | 0 | 0 (0) | 1 (0.1) |
|  | *Antimicrobial resistance, Zika virus* | 0 |  | 0 |  |  |
| N/A | *N/A* | 0 | 0 | 512 | 512 (45.1) | 512 (45.1) |
| Missing Data | *Data unavailable* | 36 | 36 (3.2) | 36 | 36 (3.2) | 72 (6.4) |
| Total |  | 1135 | 1135 (100) | 1135 | 1135 (100) |  |
